# Finding and removing highly connected individuals using suboptimal vaccines

**DOI:** 10.1186/1471-2334-12-51

**Published:** 2012-03-03

**Authors:** Beatriz Vidondo, Markus Schwehm, Andrea Bühlmann, Martin Eichner

**Affiliations:** 1 Department of Communicable Diseases, Swiss Federal Office of Public Health, Bern, Switzerland; 2 ExploSYS GmbH, Leinfelden-Echterdingen, Germany; 3 Department of Medical Biometry, University of Tübingen, Tübingen, Germany

## Abstract

**Background:**

Social networks are often highly skewed, meaning that the vast majority of the population has only few contacts whereas a small minority has a large number of contacts. These highly connected individuals may play an important role in case of an infectious disease outbreak.

**Methods:**

We propose a novel strategy of finding and immunizing highly connected individuals and evaluate this strategy by computer simulations, using a stochastic, individual-and network-based simulation approach. A small random sample of the population is asked to list their acquaintances, and those who are mentioned most frequently are offered vaccination. This intervention is combined with case isolation and contact tracing.

**Results:**

Asking only 10% of the population for 10 acquaintances each and vaccinating the most frequently named people strongly diminishes the magnitude of an outbreak which would otherwise have exhausted the available isolation units and gone out of control. It is extremely important to immunize all identified highly connected individuals. Omitting a few of them because of unsuccessful vaccination jeopardizes the overall success, unless non-immunized individuals are taken under surveillance.

**Conclusions:**

The strategy proposed in this paper is particularly successful because it attacks the very point from which the transmission network draws its strength: the highly connected individuals. Current preparedness and containment plans for smallpox and other infectious diseases may benefit from such knowledge.

## Background

Super-spreader events (cf. [1]) crucially influence the course of infectious disease outbreaks, as has been shown for SARS, measles and smallpox. Targeting control efforts on individuals with highest potential to spread disease is more effective than mass control [1]. This is very important for diseases like smallpox for which herd immunity is decreasing and stockpiled vaccines are of low eligibility or uncertain immunogenicity [2-6]. Specific information on social networks and on their contact structures is still scarce, but common properties have been revealed for many networks [7]: it has been shown that the degree distribution of social networks are frequently highly skewed, i.e. they are bound together by just a few very highly connected individuals. The frequency of contacts in such networks is not Poisson distributed (as would be expected in networks which originate from a random mixing process), but follows a skewed and long-tailed distribution [8]: the vast majority of the population has rather few contacts whereas a small minority has a huge number of contacts. Highly skewed networks are ubiquitous in nature and it seems that this particular topology confers "dynamical robustness and reliability to perform a certain function in the presence of perturbations" [9].

Indeed, a highly skewed frequency distribution of the number of contacts per person has some astonishing effects on the transmission of infection diseases and on the effect of interventions [1]. In contrast to the results with an assumption of a homogeneous mixing population, the disease transmission is only marginally reduced if a percentage of individuals is immunized at random and, thus, "removed" from the transmission network. In the presence of highly connected individuals, even the celebrated basic reproduction number *R*_0_, originally defined as "the average number of secondary cases caused by a single index case in a completely non-immune homogenously mixing population where no interventions are taken", no longer predicts whether an outbreak can occur or not [8,10-13].

These same highly-connected individuals which stabilize the transmission properties of a skewed contact network in the case of random "removal" of individuals, also make these networks vulnerable, if they can be identified and "removed". One approach to identify them, considering a theoretical infection process spreading on a skewed contact network, has been termed "acquaintance immunization" [14,15]. Here, people are picked at random and asked to name one contact each who will then be vaccinated. People with many contacts are most likely to be mentioned by somebody and are very likely to be vaccinated. An even more efficient strategy has been found by assuming that individuals can guess information about their neighbors and their contacts [16]. Acquaintances have also been shown to be good social network sensors for early detection of outbreaks [17].

Inspired by Cohen et al. [14,15], the aim of the present paper is to explore the effect of such targeted immunization strategies on the course of an epidemic considering a real disease -smallpox- using pessimistic assumptions and suboptimal vaccines. We use computer simulations based on a stochastic transmission model where individuals are connected with each other in two superimposed networks, which allows us to distinguish between close contacts (comprising family members and close friends which can easily be traced) and casual contacts who will be more worrisome in case of an outbreak, because they are more difficult to detect and to be placed under surveillance. For the latter ones, we use a highly skewed network. Our baseline intervention scenario considers case isolation (with a limited capacity) and tracing of close contacts which we combine with a pre-emptive vaccination of highly connected individuals. In our simulations, we identify highly connected individuals by first "asking" a small random fraction of the population to supply the names of their casual contacts. The most frequently mentioned contacts are then offered to be vaccinated. Depending on the simulation scenario, only a fraction of the contacts can be named, or alternatively, only a fraction of the most frequently mentioned contacts can be immunized (this may be caused by combination of a low vaccination eligibility and an imperfect vaccine efficacy). In some of the considered scenarios, highly connected individuals are additionally placed under surveillance for an indefinite duration, so that they can be prevented from spreading the infection.

## Methods

We use a stochastic, individual- and network-based simulation approach. Individuals have discrete states which can be changed by events that are scheduled on a continuous time scale and executed using a discrete event simulation algorithm. Executed events can trigger future events which affect the same individual or -through the contact network- other individuals.

### Contact network

The population consists of 100,000 fully susceptible individuals which form the nodes of a network graph. The contact network is a combination of two networks, representing two types of links: close contacts are represented by a two-dimensional toroidal square lattice with eight nearest neighbours as contacts, a so-called Moore neighbourhood; casual contacts are represented by a highly skewed network created with the Barabási-Albert algorithm that starts with a fully interconnected network of 12 nodes and then uses preferential attachment to add nodes [7]. Each individual of the population is characterized by two internal states: the infection state and the surveillance state. All individuals are 100% susceptible at the beginning of the simulation.

### Smallpox natural history

The infection model which controls the natural history of the disease is an extended SEIR-model (see Table 1 and Additional file 1: Figure S1). The simulation starts with all individuals being susceptible except for 100 randomly selected individuals who are newly infected with the virus. As their infection progresses, the infected individuals develop prodromal fever and then proceed through an early rash, a middle rash and a late rash state. A fraction of individuals dies in the early rash state, all others acquire a lasting immunity after recovery.

**Table 1:** Simulation input parameters

Population size	100,000 individuals
Index cases	100 index cases

Latency duration	12 days (gamma distributed; C.V. = 18%)

Prodromal fever duration	3 days (constant)

Early rash duration	3 days (constant)

Middle rash duration	3 days (constant)

Late rash duration	16 days (constant)

Observation duration	21 days (constant)

Case detection	unobserved cases: 2 days after onset of early rash (constant) observed cases: immediately after onset of prodromal fever

Case isolation capacity	500 units

Contact tracing finds	100% of close contacts 10% (maximum: 20 per case) of casual contacts

Case isolation prevents	100% of close contacts 100% of casual contacts

Seclusion prevents	50% of close contacts 100% of casual contacts

Case fatality ratio	30% (after early rash)

### Transmission model

A detailed description of the infection process is given in the Appendix.

### Surveillance model

The surveillance model (Additional file 2: Figure S2) controls individual states related to case detection, contact tracing, observation, and interventions like isolation or seclusion. At the start of each simulation, all individuals are unobserved. Two days after a yet unobserved new case develops the earliest signs of a rash, he or she is detected. Immediately after case detection, all close contacts and 10% of the casual contacts of the case are traced and put under observation which lasts for a maximum of 21 days (which is longer than the maximum incubation period). Observed individuals are detected immediately after developing prodromal fever. After detection, cases ought to be isolated immediately, but the number of isolation units is limited. To deal with this limited resource, all detected cases first enter a waiting queue; as soon as free isolation units become available, they are isolated (isolation is assumed to prevent all further contacts). While no free isolation units are available, detected cases are asked to seclude themselves which means that they will prevent all casual and 50% of their close contacts. Secluded cases are immediately isolated when free isolation units become available.

### Vaccination

Vaccination is implemented by first asking a fraction of the population to supply the names of their casual contacts. The most frequently mentioned contacts are then pre-emptively vaccinated, before the outbreak occurs. Technically, we first define the index cases and then define the 'vaccinated' individuals, different from those who are index cases. We do this to ensure a standard number of index cases (100), all of them unvaccinated. Depending on the simulation scenario, only a fraction of the contacts can be named, or alternatively, only a fraction of the most frequently mentioned contacts can be immunized (this may be caused by combination of a low vaccination eligibility and an imperfect vaccine efficacy). In some of the considered scenarios, vaccinated individuals are additionally placed under surveillance for an indefinite duration, so that they can be prevented from spreading the infection.

## Results

**Figure 1:**
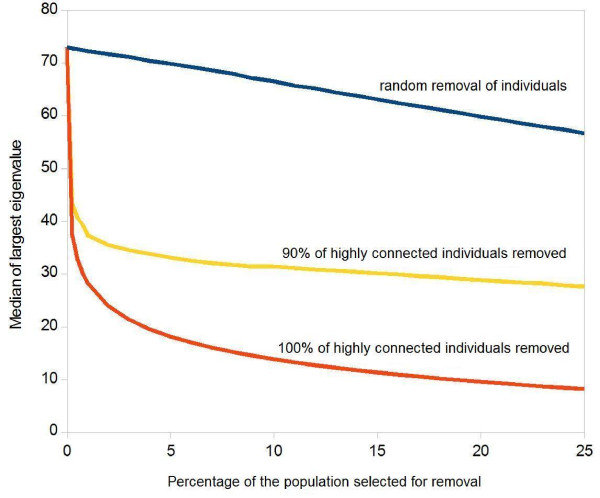
**Influence of the Removal of Highly Connected Individuals on the Maximum Eigenvalue of the Next Generation Matrix of the Remaining Casual Contact Network**. The curve shows median values of 100 simulations, given in arbitrary units. Red and yellow curves: Individuals are first sorted by their number of contacts; the removal starts with the person who has most contacts and progresses towards less highly connected ones. (**a**) Red curve: the horizontal axis shows what percentage of the population has been removed. (**b**) Yellow curve: as before, but a random sample of 90% of the selected individuals is removed. (**c**) Blue curve: people are not pre-sorted by their number of contacts, but are removed at random.

In Figure 1, we explore the theoretical limits of what can be achieved by "removing" highly connected individuals from the contact network: the vertical axis shows the maximum eigenvalue of the next generation matrix of the casual contact network which predicts the initial spread of the epidemic. The horizontal axis shows the fraction of the population which is removed from the transmission network (e.g. by immunization), whereby the individuals are sorted by their number of contacts, such that the most highly connected individuals will be removed first (red curve). Even if only a small fraction of the population is removed, Figure 1 shows a steep decline in the eigenvalue, which clearly indicates that removing the most highly connected individuals has a huge effect on the index cases' capacity to spread the infection, as has been shown in other publications (*1)*. The blue curve in Figure 1 shows a much less dramatic effect which is gained if individuals are removed at random - irrespective of their number of contacts.

**Figure 2:**
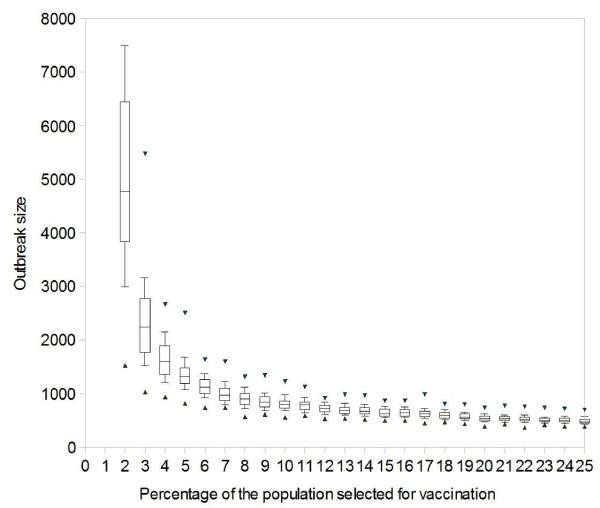
**Influence of Perfect Vaccination of Highly Connected Individuals on a Smallpox Outbreak**. Efficacy = 100%. Simulated outbreak sizes, caused by 100 index cases in a population of 100,000: the boxes show 25%, 50%, 75% quantiles, the whiskers show 10% and 90% quantiles and the triangles show minimum and maximum results (for 2% vaccination, the upper limit is not given, as one of the 100 simulations afflicted more than half of the population, as did all simulations for 0% and 1% vaccination). Targeted vaccination: (**a**) everybody is asked to name all casual contacts; (**b**) these contacts are sorted by frequency; (**c**) vaccination starts with the most frequently named person and progresses towards less frequently named ones. The horizontal axis shows what percentage of the population is vaccinated.

The relevance of this finding can be seen in Figure 2, which shows how the targeted immunization influences the simulation results of an outbreak which starts with 100 index cases in a population of 100,000 individuals. We start with a highly over-simplified situation in which we assume that we can ask everybody in the population to supply the names of all casual contacts, and later progress towards more realistic scenarios. The contacts are first sorted by frequency and then scheduled for vaccination, starting with the most frequently named person. As described in detail in the Methods section, detected cases are isolated or are asked to stay at home or to seclude themselves from contacts with other individuals (if all 500 isolation units are occupied); their contacts are traced and taken under surveillance. If only case isolation and contact tracing are used to fight against the outbreak, the large initial attack overwhelms the public health resources and the smallpox outbreak afflicts more than 50% of the population. When additionally targeting the vaccination on the most frequently named contacts, an immunization coverage of only 4% yields a median outbreak of less than 1,600 cases; a vaccination coverage of 6% can further reduce the median to less than 1,300 cases with a worst case scenario of under 1,700 cases.

**Figure 3:**
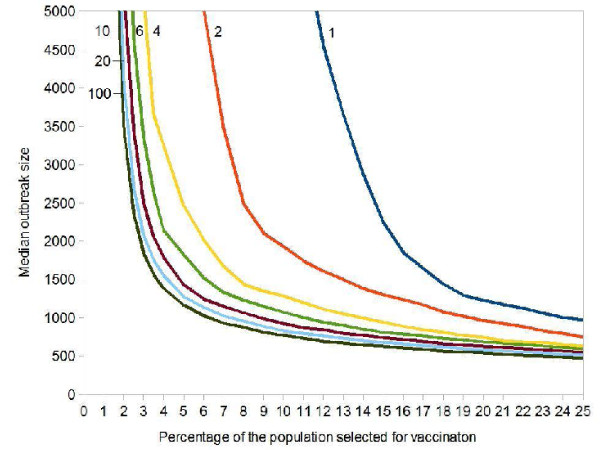
**Influence of Perfect Vaccination of Highly Connected Individuals on the Median Smallpox Outbreak Size**. Each point was obtained from 1,000 simulations. Initial value: 100 index cases in a population of 100,000. Vaccine efficacy = 100%. Targeted vaccination: (**a**) a random sample of the population (percentage is shown next to the curves) is asked to name all casual contacts; (**b**) these contacts are sorted by frequency; (**c**) vaccination starts with the most frequently named person and progresses towards less frequently named ones. The horizontal axis shows what percentage of the population is vaccinated.

Asking everybody in the population is clearly an unrealistic task for any public health system. We will next explore how the results change, if only a small random fraction of the population is asked to name all casual contacts. Figure 3 shows how the median outbreak size changes if only a small random sample is asked. The horizontal axis again shows what percentage of the population is vaccinated, whereby immunization begins with the most frequently named contact. If we are willing to vaccinate 10% of the population, it suffices to ask a random sample of 4% of the population for their contacts in order to obtain a median outbreak size of less than 2,000 cases. For the highly optimistic goal of having a median outbreak size of less than 1,000 cases, we have to ask 10% of the population to supply the names of their contacts.

**Figure 4:**
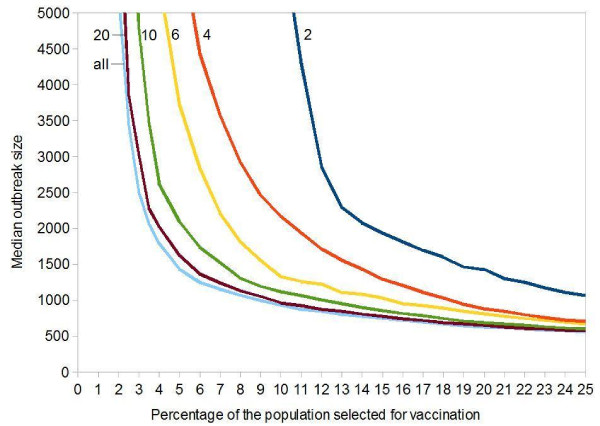
**Influence of Perfect Vaccination of Highly Connected Individuals on the Median Smallpox Outbreak Size**. Each point was obtained from 1,000 simulations. Initial value: 100 index cases in a population of 100,000. Vaccine efficacy = 100%. Targeted vaccination: (**a**) a random sample of 10% of the population is asked to name casual contacts each (number of contacts asked is given next to the curves); (**b**) these contacts are sorted by frequency; (**c**) vaccination starts with the most frequently named person and progresses towards less frequently named ones. The horizontal axis shows what percentage of the population is vaccinated.

In reality, hardly anybody may be able to recall all casual contacts. In the next refinement, we assume that people are asked to supply only a limited number of casual contacts. Figure 4 shows that an immunization of 10% of the population still yields very optimistic results, even if the 10% randomly sampled individuals only name 6 to 10 contacts each.

**Figure 5:**
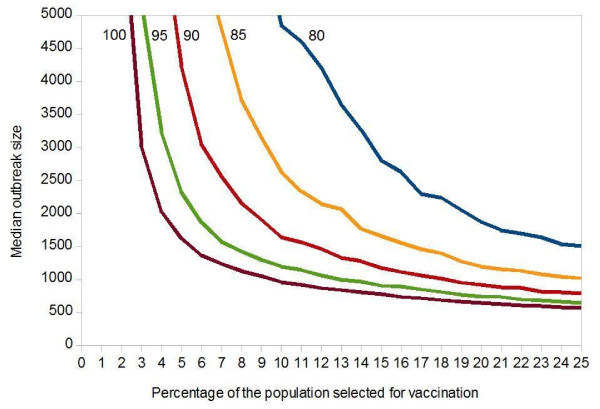
**Influence of Imperfect Vaccination of Highly Connected Individuals on the Median Smallpox Outbreak Size**. Each point was obtained from 1,000 simulations. Initial value: 100 index cases in a population of 100,000. Targeted vaccination: (**a**) a random sample of 10% of the population is asked to name 20 casual contacts each; (**b**) these contacts are sorted by frequency; (**c**) the most frequently named person is first scheduled for vaccination, less frequently named ones follow. The horizontal axis shows what percentage of the population is scheduled for vaccination. Because of vaccination eligibility and/or vaccination efficacy, only a fraction of those scheduled for vaccination can successfully be immunized (percentage immunized is given next to the curves).

In the previous scenarios, we have assumed a vaccine with 100% efficacy and we have assumed that every contact which was scheduled for vaccination was also eligible, but these assumptions cannot be made for the existing smallpox vaccines [2]. Figure 5 explores the influence of an imperfect vaccine on the median of the outbreak size.

Successfully immunizing as much as 80% or 90% of the scheduled individuals may only be possible with one of the new generation vaccines [2-6], and even this may already be regarded as over-optimistic by some, yet this assumption already increases the median outbreak size considerably (Figure 5). The disproportionate effect of a few vaccination failures on the simulation result can be explained by a closer look at the maximum eigenvalues depicted in Figure 1: The removal of all highly connected individuals (red curve) is necessary to really incapacitate the transmission network; if only a random sample of 90% of the highly connected individuals are removed (yellow curve), it practically stays intact.

**Figure 6:**
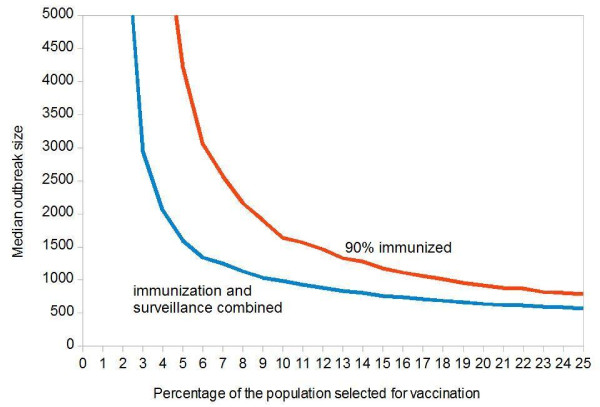
**Combined Influence of Imperfect Vaccination and Surveillance of Highly Connected Individuals on the Median Smallpox Outbreak Size**. Each point was obtained from 1,000 simulations. Initial value: 100 index cases in a population of 100,000. Targeted vaccination: (**a**) a random sample of 10% of the population is asked to name 20 casual contacts each; (**b**) these contacts are sorted by frequency; (**c**) the most frequently named person is first scheduled for vaccination, less frequently named ones follow. The horizontal axis shows what percentage of the population is scheduled for vaccination. Because of vaccination eligibility and/or vaccination efficacy, a fraction of 90% (red curve) or 100% (blue curve) are successfully immunized. Scheduled individuals who are not immunized are permanently taken under surveillance.

In our final scenario, we combine immunization targeted to highly connected individuals (using an imperfect vaccine) with the surveillance of the vaccinated individuals: each contact which is scheduled for vaccination, will be taken under surveillance (if the vaccination success can be determined, it is sufficient to observe contacts with failed vaccination and contacts who were excluded from vaccination). Figure 6 assumes that a random sample of 90% of the individuals who are scheduled for vaccination can really be immunized. The blue curve shows the median outcome with, the red curve without additional surveillance of selected individuals.

## Discussion

Our results show that a combination of a novel strategy of finding and immunizing highly connected individuals with case isolation and contact tracing can prevent a large smallpox outbreak, with the advantage of vaccinating only about 10% of the population (Figure 2). This low vaccination coverage considerably reduces the number of severe side effects and deaths due to vaccination [2,3,18]. In the case of smallpox vaccines this is of paramount importance, as reported by Casey et al. 2006, "after the inoculation of 37,901 people in the United States, three deaths, two permanent disabilities, and ten life-threatening illnesses were attributed to vaccination during 2003" [6].

Successful control strategies proposed for an intentional release of smallpox, vary from targeting high-risk individuals to broader random vaccination campaigns and no one control method can be identified a priori as best [19-22]. Uncertainty with respect to transmission before the onset of symptoms, residual herd immunity, demographics and mobility exist, so their differing conclusions can largely be attributed to underlying differences in model structures and parameter assignments. Our study is the first that uses a highly skewed contact structure for a smallpox outbreak.

In comparison to previous modeling studies, and in order to show the strength of this strategy, we focus on a highly pessimistic scenario: (a) the outbreak starts with the simultaneous infection of 100 independent index cases on a fully susceptible population; (b) a high proportion of transmission takes place during the prodromal fever phase which does not yet reveal the nature of the infection and which does not trigger case detection except for suspects who are already under surveillance; (c) we consider a limited number of isolation units which are easily exhausted by a major outbreak; and (d) we consider suboptimal vaccines.

We have made a big effort to design a realistic model and to use plausible parameter values, yet we made a number of inevitable simplifying assumptions on aspects that we consider out of the scope of the present paper. Worth of mention is that we use a pre-assigned and bidirectional contact network, to which we superimpose a stochastic transmission process (see Appendix). The contact network constitutes 'potential contacts' and the actual transmissions occur according to the transmission process. In reality, contact structures change over time so it is uncertain whether all pre-emptively identified highly connected individuals would play a role in a future outbreak. Specially in acts of bioterrorism, super-spreading events (cf. [1]) might be difficult to foresee unless awareness is increased. We assume, however, that highly connected individuals would have a potential for disease spread and constitute a good first guess.

Our study is the first that addresses the problem of suboptimal vaccines in the case of smallpox. We show that a network- based vaccination strategy strongly depends on a very high vaccine efficacy and on a high eligibility and compliance of the people selected for vaccination. It can quickly lose its effect if some of the most highly connected individuals cannot be vaccinated or if vaccination fails protect them (Figure 5). If only a few of them are left unprotected, they can fuel a super-spreading event (cf. [1]) multiplying the infection in the population.

Adopting the described containment strategy may require to overcome political, social and ethical hurdles. A vaccination campaign against smallpox will not be initiated anywhere before a strong suspicion of bioterror attack occurs or the appearance of smallpox cases inside or outside a country has been confirmed. As long as a smallpox bioterror attack is regarded to be highly unlikely, most people would not accept to receive a potentially harmful smallpox vaccination anyway. This perception may change drastically after smallpox have reappeared somewhere in the world. Individuals who have been identified to be under the highest risk of contracting the infection may gladly accept to be chosen to receive a protective vaccine. Yet, due to their health condition, not all of them may be eligible for vaccination. As it is very important to remove all potential highly infectious individuals (Figure 5), highly connected individuals who cannot be vaccinated or whose immunization has failed must be taken under surveillance (Figure 6).

Considering the described containment strategy in preparedness plans would shift their focus to identifying potential highly infectious individuals and to preparing for pre-emptive vaccination of a pre-selected fraction of the population. It would also put emphasis on the availability of isolation units and of people trained in contact tracing and surveillance. If the public health system is overtaxed by an outbreak despite this targeted vaccination, containment strategies involving ring vaccination or large-scale vaccination should be implemented. Public health emergency plans have to provide instructions on (1) the observation of the dynamics of an outbreak and (2) which observations trigger the transition from a targeted containment strategy to a population-based one.

Infections which share the same route of transmission may also have the same highly connected individuals. Knowing which individuals have many contacts (whereby a "contact" depends on the mode of transmission of an infectious agent) may help public health agencies to control whole groups of infectious diseases, including newly emerging ones, rather than individual diseases. In specific settings, highly connected individuals may be identified using other approaches: in hospitals and companies, an analysis of the team structure and its meeting schedules can hint at who has most contacts. This may also affect the focus of business continuity plans, written to prepare for pandemic influenza or similar events. Simulation studies show that highly connected individuals are reached rather early by newly introduced infections. Thus, the knowledge of such people can also be used to improve outbreak detection [17].

## Conclusions

Current preparedness and containment plans for smallpox and other directly transmitted diseases like measles and pandemic influenza can benefit from considering the described pre-emptive strategy which effectively targets the fraction of the population fuelling disease spread and minimizes vaccine related side effects.

## Competing interests

The authors declare that they have no competing interests.

## Authors' contributions

BV developed the hypothesis, participated in the model design and the discussions of the scenarios, wrote the manuscript, and coordinated the project. MS implemented the software and designed the model, produced the simulation results and figures, and participated in writing the manuscript. AB participated in writing the manuscript and in the discussions of the scenarios depicted in the model. ME designed the mathematical model, participated in the discussions of the scenarios, and wrote the manuscript. All authors read and approved the final manuscript.

## Appendix: Details on the infection process

We define eight expected numbers *E_s,n _*of secondary infections per index case which depend on the index case's infectious stage *s *∈ {prodromal, earlyrash, middlerash, laterash} and on the network *n *∈ {close, casual} in which the secondary cases are produced (Table S1). This distinction enables us to consider a more intense exposure of close contacts and to modify the cases' contagiousness in their different disease stages, as suggested by previous smallpox studies [19,23]. We set the sum of these expected values to 5, the estimate at smallpox's eradication [24]. In a homogeneously mixing population, this would be called the basic reproduction number *R*_0_. We have decided to parameterize our model with these expected values rather than the maximum eigenvalue of the next generation matrix, not only because expected values are more intuitive, but also because estimates of *R*_0 _are frequently based on secondary cases [14,23] or on homogeneous mixing assumptions [25,26]. As the maximum eigenvalue better predicts the initial spread of an epidemic, we use it in Figure 3 in the results. In the simulation model, we implement the following infection process: when an individual enters one of the four contagious states, it triggers future infection events for each of its contacts. This is done in the following way: (1) we chose the effective contact rate *β_s,n _*such that

(1) $$\beta_{s,n}\int_{0}^{D_{s}}e^{-\beta_{s,n}t}dt=\frac{E_{s,n}}{c_{n}},$$

where *D_s _*is the duration of the specific contagious stage s and *c_n _*is the average number of contacts per person in network *n *(for close contacts, *c_n _*is 8, for casual ones, it is 24). Solving Equation 1 yields

(2) $$\beta_{s,n}=\frac{-\ln(1-E_{s,n}/c_{n})}{D_{s}}$$

(2) we draw a random number *r *from an exponential distribution with mean 1/*β_s,n_*, (3) if *r *is smaller than the duration of the current contagious state *D_s_*, we schedule an infection event for the corresponding contact with delay *r*, otherwise the infection event is discarded. Infection events are stored in an event queue. At the scheduled time, it is checked whether the infection source is still contagious, the prospective victim is still susceptible and the contact between the two has not yet been impeded by interventions (e.g. isolation or seclusion) (see Table 1). The infection only takes place if all of these three conditions are fulfilled.

**Table 2:** Expected Number of Secondary Cases per Index Case by Disease Stage and Contact Type

Disease stage *s*	Expected number of secondary cases *E_s,n_*	
	**in the close network** **(toroidal grid; 8 contacts constant)**	**in the casual network** **(Barabási-Albert highly skewed network starting with 12 contacts; average: 24)**

Prodromal fever	0.54	0.54

Early rash	2.39	0.80

Middle rash	0.21	0.07

Late rash	0.34	0.11

## Pre-publication history

The pre-publication history for this paper can be accessed here:

http://www.biomedcentral.com/1471-2334/12/51/prepub

## Supplementary Material

Additional file 1 **Figure S1**. Infection model. Click here for file

Additional file 2 **Figure S2**. Surveillance model. Click here for file
