# Supplementary figures and images for: Finding and removing highly connected individuals using suboptimal vaccines
Source: BMC Infect Dis. 2012 Mar 3;12:51. doi: 10.1186/1471-2334-12-51 (PMC3316139; doi:10.1186/1471-2334-12-51)

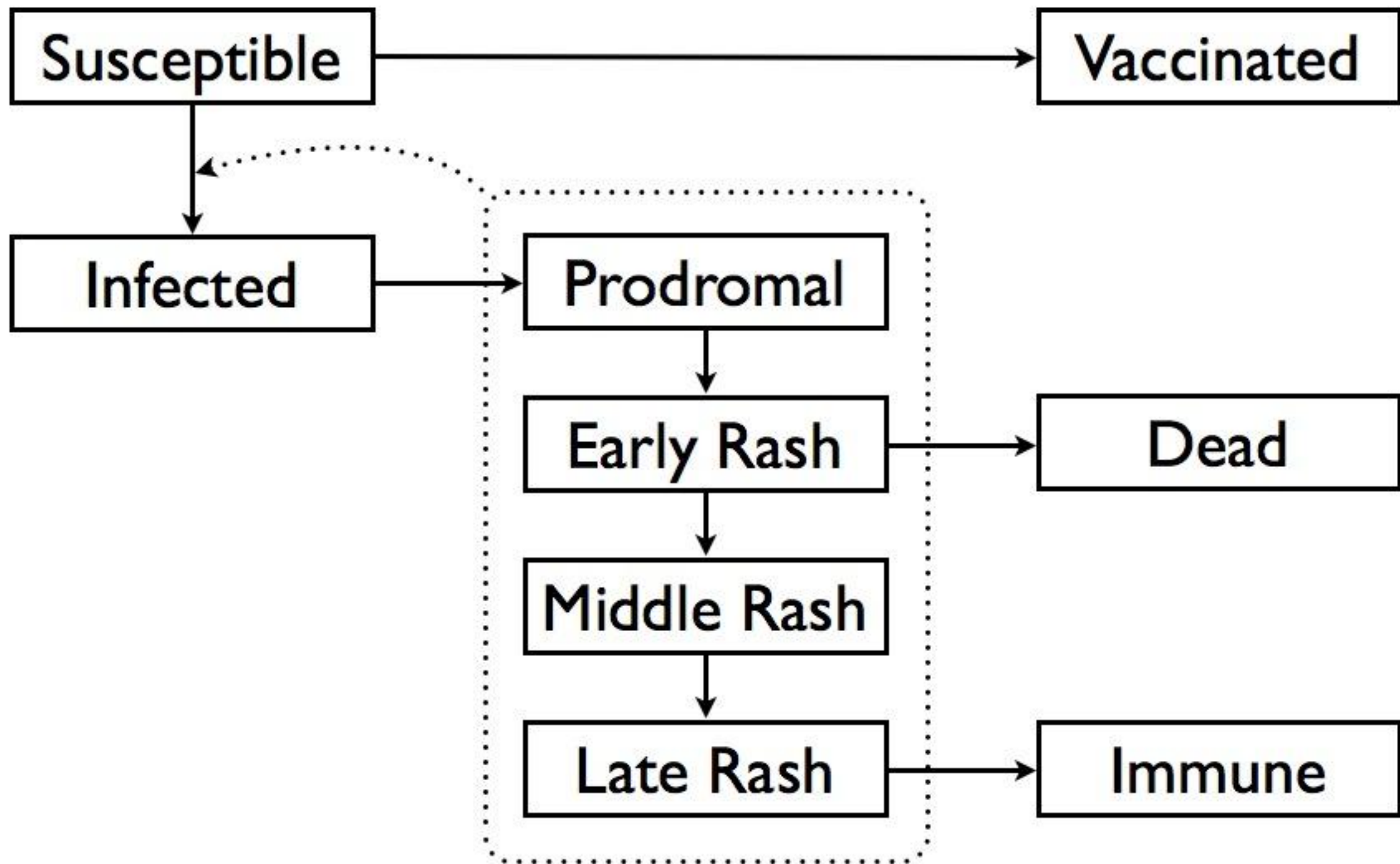

Supplement: Additional file 1 — Figure S1. Infection model. [file 1471-2334-12-51-S1.PDF]

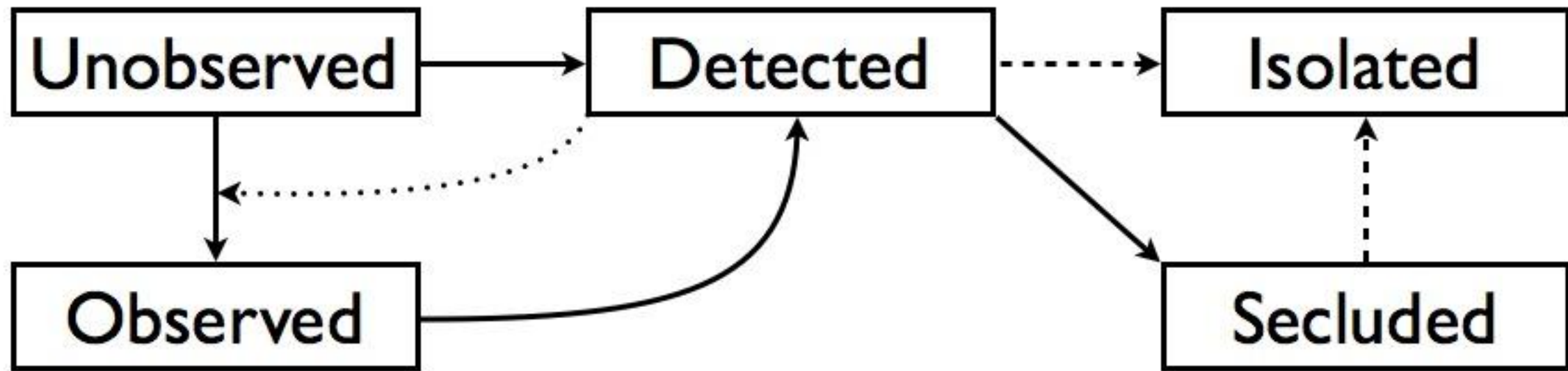

Supplement: Additional file 2 — Figure S2. Surveillance model. [file 1471-2334-12-51-S2.PDF]
